# Evaluation of Small Intestine Grafts Decellularization Methods for Corneal Tissue Engineering

**DOI:** 10.1371/journal.pone.0066538

**Published:** 2013-06-14

**Authors:** Ana Celeste Oliveira, Ingrid Garzón, Ana Maria Ionescu, Victor Carriel, Juan de la Cruz Cardona, Miguel González-Andrades, María del Mar Pérez, Miguel Alaminos, Antonio Campos

**Affiliations:** 1 Tissue Engineering Group, Department of Histology, University of Granada, Granada, Spain; 2 Department of Optics, University of Granada, Granada, Spain; 3 Division of Ophthalmology, University of Granada, Granada, Spain; University of Reading, United Kingdom

## Abstract

Advances in the development of cornea substitutes by tissue engineering techniques have focused on the use of decellularized tissue scaffolds. In this work, we evaluated different chemical and physical decellularization methods on small intestine tissues to determine the most appropriate decellularization protocols for corneal applications. Our results revealed that the most efficient decellularization agents were the SDS and triton X-100 detergents, which were able to efficiently remove most cell nuclei and residual DNA. Histological and histochemical analyses revealed that collagen fibers were preserved upon decellularization with triton X-100, NaCl and sonication, whereas reticular fibers were properly preserved by decellularization with UV exposure. Extracellular matrix glycoproteins were preserved after decellularization with SDS, triton X-100 and sonication, whereas proteoglycans were not affected by any of the decellularization protocols. Tissue transparency was significantly higher than control non-decellularized tissues for all protocols, although the best light transmittance results were found in tissues decellularized with SDS and triton X-100. In conclusion, our results suggest that decellularized intestinal grafts could be used as biological scaffolds for cornea tissue engineering. Decellularization with triton X-100 was able to efficiently remove all cells from the tissues while preserving tissue structure and most fibrillar and non-fibrillar extracellular matrix components, suggesting that this specific decellularization agent could be safely used for efficient decellularization of SI tissues for cornea TE applications.

## Introduction

Tissue engineering (TE) is an interdisciplinary field that applies the principles and methods of engineering to the development of biological substitutes that can restore, maintain, or improve tissue formation [1] by combining cells, scaffolds and suitable growth factors.

Numerous corneal disorders commonly lead to end-organ failure and require corneal transplantation. However, the lack of donors and the risk of graft immune rejection represent critical limitations of this approach. In recent years, the development of cornea substitutes by TE emerged as an alternative method to overcome the complications of corneal transplantation, and different corneal substitutes have been developed using fibrin-agarose [2]–[5], fish scales [6], chitosan [7], caprolactone [8], collagen [9], poly (ester urethane) urea [10] and other biomaterials. However, new scaffolds are in need for the generation of more efficient cornea substitutes by TE. In this regard, decellularization has been reported as an efficient method for the generation of biologic scaffolds for TE applied to the kidney [11], lung [12], cornea [13], liver [14] and heart [15]. Decellularization protocols are focused on the removal of all cellular material without adversely affecting the composition, biological activity, or mechanical integrity of the remaining three-dimensional matrix [16].

A number of physical, chemical and enzymatic methods have been described for tissue decellularization [17]. However, none of these methods is suitable for all applications, and each specific tissue or organ may require the use of different decellularization agents and different decellularization protocols. The most effective agents for decellularization of each tissue and organ may depend on many factors, including the tissue cellularity (e.g. liver vs. tendon), density (e.g. dermis vs. adipose tissue), lipid content (e.g. brain vs. urinary bladder) and thickness (e.g. dermis vs. pericardium) [18].

One of the most commonly decellularized organs is the gastrointestinal tract. In fact, decellularized porcine small intestine have been used clinically for reconstruction of distinct human organs, including the lung [19], urethra [20] and vagina [21]. Probably, the use of these materials for clinical purposes is a consequence of their content of a number of growth factors and cytokines such as fibroblasts growth factors (FGF), transforming growth factor-beta (TGF-β), structural and functional proteins that are part of native mammalian extracellular matrix (ECM) [22]. Although the role of these molecules in tissue engineering of the human cornea is not well known, several research works demonstrated that the normal cornea contains some of these relevant ECM components and that their presence may be necessary for the correct differentiation of cornea cells [23]. Additionally, several publications previously demonstrated that the cornea can be efficiently reconstructed in cats, dogs and horses [24]–[26] by using porcine small intestine grafts. The ECM scaffold derived from decellularized porcine intestine has been extensively characterized for different purposes [27]. However, the use of this scaffold to reconstruct a cornea has not been deeply explored and standardized until now.

The objectives of the present work were (1) to decellularize the small intestine by using several chemical and physical methods, and (2) to evaluate the effectiveness of cells removal, ECM components preservation (glycoproteins, proteoglycans, collagen and reticular fibers), and the optical properties of the decellularized tissues.

## Methods

### Preparation

Fresh small intestines (SI) were obtained from adult mice (n = 7) euthanatized under general anesthesia. Then, SI were carefully washed in phosphate buffered saline (PBS) to completely remove all the intestinal contents, and 1.5 cm-length fragments were obtained from each SI for immediate decellularization (7 samples per decellularization protocol). Samples were weighted before and after decellularization to determine the percentage of tissue weight removal. All experiments were conducted in accordance with animal care protocols of the ethics and animal research committees at the University of Granada, Spain. Native non-decellularized SI fragments were used as control.

### Decellularization Protocols

#### Chemical methods

For decellularization with chemical agents, samples were first immersed in one of the following aqueous solutions: NaCl (1.5 M, 3 M, 5 M), triton X-100 (0.1%, 0.3%, 0.6%), or SDS (0.1%, 0.3% or 0.6%) for 24 h. Then, all specimens were rinsed three times in PBS, and immersed again in the same freshly-prepared decellularization solution for 24 h. Finally, samples were washed with PBS three times. Chemical treatments were performed at room temperature (R/T) and under continuous soft agitation. All protocols were performed by using aqueous solution to avoid a loss of water that could cause changes in the tissue architecture as previously suggested [27].

#### Physical methods

For decellularization by physical protocols, samples were immersed in a Petri dish with PBS buffer and subjected to sonication (SC) or ultraviolet (UV) treatment. Specimens corresponding to the SC experimental group were sonicated for 5 min, 10 min or 15 min in an ultrasonic emissor (Fungilab S.A). UV samples were exposed to UV light of 254 nm wavelength for 5 min, 10 min or 15 min using a 15 W ultraviolet lamp (Havells Sylvania) at a working distance of 15 cm. Then, the procedure was repeated during identical periods of time, but on the opposite tissue side. After SC or UV treatment, samples were rinsed three times with PBS. All physical treatments were carried out at R/T.

### Histological and Histochemical Analyses

Fresh control and decellularized SI samples were fixed in 10% buffered formalin and embedded in paraffin, and 5 µm-thick sections were obtained. For semi-quantitative analysis of tissue structure, deparaffinized sections were stained with hematoxylin and eosin (HE) and analyzed using a light microscope (Nikon Eclipse 90i). The following classification scale was used: (0) highly organized tissue, (1) low levels of disorganization, (2) intermediate levels of disorganization, (3) high tissue disorganization. Illustrative examples for this scale are shown in Fig. S1. Two different sections corresponding to each SI fragment were analyzed (n = 14 per experimental group), and all tissue sections were classified by the same histologist. Then, the tissue structure was analyzed by scanning electron microscopy (SEM). For this, fresh control and decellularized SI samples were fixed in 2.5% glutaraldehyde, dehydrated in increasing acetone series (30%, 50%, 70%, 95%, and 100%), critical point dried, mounted on aluminum stubs, sputter coated with gold and examined in a scanning electron microscope (Quanta 200; FEI, Eindhoven, The Netherlands). In each sample, two parameters were evaluated: 1) size of the interfibrillar spaces, and 2) three-dimensional structure and organization of the tissue collagen fibers. The following classification scale was used for the interfibrillar spaces: (−) small and regular interfibrillar spaces, (+) medium-size interfibrillar spaces, (++) large interfibrillar spaces, (+++) very large and irregular interfibrillar spaces. For the three-dimensional structure and organization of the tissue collagen fibers, we used the following scale: (+++) highly organized fibers and adequate three-dimensional structure, (++) partially organized fibers and moderately altered three-dimensional structure, (+) disorganized fibers and altered three-dimensional structure, (−) highly disorganized fibers and disrupted three-dimensional structure. Two tissue fragments corresponding to each SI fragment were analyzed (n = 14 per experimental group).

To determine the effectiveness of cell removal (decellularization efficiency), two different analyses were carried out. First, the number of remaining nuclei in control and decellularized tissues was quantified using 4,6-diamidino-2-phenylindole (DAPI) staining on deparaffinized tissue sections, and the percentage of cell removal was determined for decellularized samples. Then, total residual DNA was quantified in each tissue. With that purpose, tissue DNA was extracted and purified using the QIAgen DNeasy Blood & Tissue Kit and DNA was quantified by using Quant-iT™ PicoGreen (Invitrogen, Carlsbad, CA, USA). Three tissue samples were analyzed for the 7 SI fragments included in each experimental group for both DAPI and DNA quantification (n = 21 per experimental group).

For quantitative analysis of the ECM structure, the staining intensity level of collagen, reticular fibers, glycoproteins and proteoglycans was determined on tissue sections stained with picrosirius, Gomori reticulin, periodic acid-Schiff (PAS) and alcian blue, respectively. Briefly, 5 µm-thick tissue sections were deparaffinized and rehydrated to distilled water. Then, the following protocols were followed:

To evaluate the presence of collagen fibers, Picrosirius staining was performed using Picrosirius working solution (Sirius red F3B) for 30 min and counterstained with Harris’s Hematoxylin for 5 min.
To analyze reticular fibers, the Gomori’s reticulin metal reduction method was applied using 1% potassium permanganate for 1 min, followed by 2% sodium metabisulphite solution and sensibilization with 2% iron alum for 2 min. Then, ammoniacal silver was used for 10–15 min and 20% formaldehyde for 3 min. Differentiation was performed with 2% gold chloride for 5 min and 2% thiosulphate for 1 min. Nuclear fast red was used for 1 min as counterstaining agent.
To assess glycoproteins content, the Periodic acid-Schiff stain method (PAS) was used. 0.5% periodic acid solution was used for 5 min as oxidant, followed by incubation in Schiff reagent for 15 min and counterstaining with Harris’s Haematoxylin for 1 min.
To determine proteoglycans content, samples were incubated in Alcian blue solution for 30 min and counterstained with nuclear fast red solution for 1 min.

For each experimental group, histochemistry for a specific component was performed at the same time for all samples, using the same reagents during the same time to ensure reproducibility of the results. Then, histological images were taken using 200X magnification by using a light microscope (Nikon Eclipse 90i) and the intensity of the staining of each specific ECM component was quantified by using ImageJ software as previously reported [28]. All images were taken and analyzed using exactly the same conditions (exposition time, white balance, background, etc.) with the Nikon NIS-Elements software. For each image corresponding to each experimental group, 10 small areas (n = 70) were randomly selected at the area of interest (connective tissue ECM) and the staining intensity was automatically calculated by the program, and subtracted to the background blank signal.

### Analysis of Optical Properties

First, gross transparency of each sample was assessed by placing the tissues on a patterned surface to visually estimate how transparent they were. Then, the spectral distribution of the transmitted light through the decellularized SI samples and also through control samples and a native porcine cornea was determined using a Helios Alpha UV-Vis Spectrophotometer (Thermo Electron Corporation, USA). The transmitted light was calculated in the 400–700 nm spectral range as 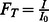, where *_I_* is the transmitted light intensity and 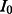 is the incident light intensity. Thickness was calculated for each sample by using a Nikon Eclipse 90i light microscope, and the results were normalized for equivalent tissue thicknesses.

### Statistical Analysis

In this work, we used five different decellularization agents (NaCl, SDS, triton X-100, SC and UV), with three different specific conditions for each agent (different concentrations for chemical methods and different times for physical methods). Then, we determined the average or median values and the standard deviation or quartiles of the following experimental groups: 1) all samples treated with the same decellularization agent independently of the concentration or the time used. These groups were named as global groups. 2) Samples treated with a specific concentration or time of each agent.

To perform statistical analysis, we first confirmed the normality of each distribution using the Kolmogorov-Smirnov statistical test. Then, all normally-distributed global groups (DAPI, DNA quantification, sample weight, picrosirius, Gomori reticulin, PAS, alcian blue and light transmittance) were compared with control non-decellularized tissues and with the rest of experimental global groups by using Student *t* test. For non-normal distributions (HE), the non-parametric test of Mann-Whitney was used. Once the global differences were detected, we used the same statistical tests to compare each specific group with the control and to compare the different conditions corresponding to the same decellularization agent (i.e., 0.1% SDS vs. 0.3% SDS, 0.1% SDS vs. 0.6% SDS and 0.3% SDS vs. 0.6% SDS).

Results corresponding to normal distributions were expressed as means plus/minus standard deviations (mean±SD) and non-normal variables were expressed as medians and quartiles 1 and 3 (median, Q1–Q3). Statistically significant differences were defined as *p*<0.001 using the Bonferroni *p* value correction for multiple testing, since more than 50 different statistical comparisons were simultaneously performed in this work. P values equal to 0.001 were considered as marginally significant.

## Results

### Efficiency of the Decellularization Protocols

**Figure 1:**
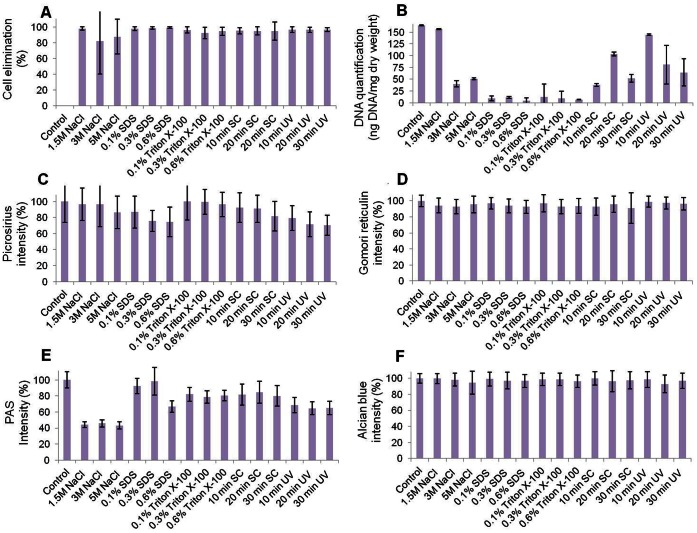
Histograms representing the efficiency of cell removal and tissue structure preservation as determined by DAPI (A), residual DNA quantification (B), picrosirius collagen staining (C), Gomori reticulin (D), PAS glycoprotein staining (E) and alcian blue proteoglycan staining (F). Bars correspond to average values ± standard deviations.

In the first place, the cell removal efficiency as determined by DAPI staining (Fig. 1A and Fig. S2) revealed that all global and specific groups were significantly different to control non-decellularized SI (p<0.001). When global groups were compared to one another, we found that the more efficient global group was SDS (98.88±1.62%), which was significantly more effective (p<0.001) than the global groups triton X-100 (94.63±5.85%), SC (95.18±7.53%) and UV (96.68±3.13%), and marginally different (p = 0.001) to the NaCl global group (89.50±27.81%) (Tables 1 and 2). No statistical differences were found among the specific conditions corresponding to the same decellularization agent, although the specific group that tended to show the highest decellularization percentages was the 0.6% SDS group (99.53±0.74%).

**Table 1:** Average results of the evaluation of decellularization efficiency, semi-quantitative analysis of tissue structure and quantitative analysis of fibrillar and non-fibrillar ECM components in control non-decellularized intestine and in all decellularization global groups.

Global groups			Control	NaCl	SDS	Triton X-100	SC	UV
**Decellularization efficiency**	**Nuclear cell removal (DAPI)**		0	89.50±27.81	98.88±1.62	94.63±5.85	95.18±7.53	96.68±3.13
	**Tissue DNA quantification**		164.46±1.27	82.48±3.37	8.63±4.06	9.70±14.05	64.46±5.08	96.75±23.94
**Semi-quantitative analysis of tissue structure**	**Haematoxylin-Eosin (HE)**		0	1.00;0.00–2.00	2.00;1.00–3.00	1.00;0.00–2.00	2.00;1.00–2.00	1.00;1.00–2.00
	**SEM**	**Interfibrillar spaces**	−	+	+	+	+ +	+++
		**Collagen fibers**	+++	+	+	+ +	+	−
**Quantitative analysis of ECM components**	**Picrosirius staining**		100	92.24±25.36	78.88±22.18	100.27±19.10	87.57±21.11	73.46±19.72
	**Gomori reticulin staining**		100	94.56±10.26	94.69±8.55	94.91±10.50	93.77±14.64	98.03±7.53
	**PAS staining**		100	44.19±9.50	84.34±14.06	80.47±9.67	82.20±15.98	66.48±12.89
	**Alcian blue staining**		100	97.51±10.49	97.74±9.53	98.05±8.03	98.35±11.48	96.27±10.77

Nuclear cell removal and the staining signal for picrosirius, Gomori reticulin, PAS and Alcian blue correspond to percentages in reference of control non-decellularized intestine samples and are expressed as means plus/minus standard deviations. Tissue DNA quantification is shown as nanograms of DNA per milligram of dry weight of tissue. HE results are shown as median values and quartiles Q1–Q3 and correspond to the following classification: (0) highly organized tissue, (1) low levels of disorganization, (2) intermediate levels of disorganization, (3) high tissue disorganization. The analysis of tissue structure by SEM (interfibrillar spaces and collagen fibers) is shown in a scale ranging between (−) (small and regular interfibrillar spaces and highly disorganized fibers and disrupted three-dimensional structure) and (+++) (very large and irregular interfibrillar spaces and highly organized fibers and adequate three-dimensional structure) as defined in the methods section.

**Table 2:** Statistical *p* values corresponding to the comparison of the results obtained for each decellularization method and for each technique.

COMPARISON GROUPS		DAPI	DNA	HE	Picrosirius	Gomori Reticulin	PAS	AlcianBlue	Transmittance
**GLOBAL GROUPS**	NaCl vs. Control	**0.0000^a^**	0.0049	**0.0000^a^**	0.091	**0.0000^a^**	**0.0000^a^**	0.0307	**0.0000^a^**
	SDS vs. Control	**0.0000^a^**	**0.0001**	**0.0000**	**0.0000^a^**	**0.0000**	0.0781	0.0444	**0.0000^a^**
	Triton X-100 vs. Control	**0.0000^a^**	**0.0001^a^**	**0.0000^a^**	0.9506	**0.0000^a^**	0.026	0.0744	**0.0000^a^**
	SC vs. Control	**0.0000^a^**	**0.0000^a^**	**0.0000^a^**	0.0072	**0.0000^a^**	0.051	0.1753	**0.0000^a^**
	UV vs. Control	**0.0000^a^**	**0.0006^a^**	**0.0000^a^**	**0.0000^a^**	0.0718	**0.0006^a^**	0.0018	**0.0000^a^**
	NaCl vs. SDS	**0.0010^b^**	0.0075	0.0737	**0.0000^a^**	0.8689	**0.0000^a^**	0.7737	**0.0003^a^**
	NaCl vs. triton X-100	0.1086	0.0081	0.6941	0.0012	0.6705	**0.0000^a^**	0.4709	**0.0000^a^**
	NaCl vs. SC	0.0822	0.4557	0.1721	0.0809	0.4581	**0.0000^a^**	0.3699	0.1957
	NaCl vs. UV	0.0236	0.6269	0.7127	**0.0000^a^**	**0.0000^a^**	**0.0000^a^**	0.1529	0.0046
	SDS vs. triton X-100	**0.0000^a^**	0.47	0.1703	**0.0000^a^**	0.7695	0.3306	0.6593	0.0109
	SDS vs. SC	**0.0003**	**0.0001^a^**	0.4922	**0.0007^a^**	0.3744	0.6698	0.4993	0.051
	SDS vs. UV	**0.0000^a^**	**0.0001^a^**	0.1006	0.0257	**0.0000^a^**	**0.0003^a^**	0.0771	0.1946
	Triton X-100 vs. SC	0.6333	**0.0001^a^**	0.3739	**0.0000^a^**	0.2927	0.6928	0.7317	0.0016
	Triton X-100 vs. UV	0.0078	**0.0001^a^**	0.9317	**0.0000^a^**	**0.0000^a^**	**0.0009^a^**	0.0238	**0.0001^a^**
	SC vs. UV	0.1447	0.0731	0.274	**0.0000^a^**	**0.0000^a^**	0.0026	0.0314	0.2409
**SPECIFIC GROUPS**	1.5 M NaCl vs. Control	**0.0000^a^**	0.8733	0.0115	0.5281	**0.0001^a^**	**0.0000^a^**	0.782	**0.0000^a^**
	3 M NaCl vs. Control	**0.0000^a^**	**0.0002^a^**	0.0174	0.5065	**0.0000^a^**	**0.0000^a^**	0.1775	**0.0000^a^**
	5 M NaCl vs. Control	**0.0000^a^**	**0.0001^a^**	**0.0003^a^**	0.0075	0.0052	**0.0000^a^**	0.0018	**0.0000^a^**
	1.5 M NaCl vs. 3 M NaCl	0.0574	**0.0010^b^**	0.9684	0.9656	0.3151	0.7357	0.1784	**0.0000^a^**
	1.5 M NaCl vs. 5 M NaCl	0.0228	**0.0010^b^**	0.3637	0.0227	0.3298	0.7126	0.0011	**0.0000^a^**
	3 M NaCl vs. 5 M NaCl	0.5277	0.0107	0.3068	0.0268	0.0531	0.5069	0.0229	**0.0000^a^**
	0.1% SDS vs. Control	**0.0000^a^**	**0.0001^a^**	0.0115	0.0105	0.0308	0.4515	0.5182	**0.0000^a^**
	0.3% SDS vs. Control	**0.0000^a^**	**0.0000**	**0.0001^a^**	**0.0000^a^**	**0.0000^a^**	0.855	0.0754	**0.0000^a^**
	0.6% SDS vs. Control	**0.0000^a^**	**0.0001^a^**	**0.0001^a^**	**0.0000^a^**	**0.0000^a^**	0.0011	0.0094	**0.0000^a^**
	0.1% SDS vs. 0.3% SDS	0.234	0.5257	0.0494	**0.0004^a^**	0.006	0.5293	0.2076	**0.0000^a^**
	0.1% SDS vs. 0.6% SDS	0.01	0.0087	0.0723	**0.0008^a^**	**0.0001^a^**	**0.0002^a^**	0.0367	**0.0000^a^**
	0.3% SDS vs. 0.6% SDS	0.0259	0.098	0.7493	0.7548	0.3449	**0.0001^a^**	0.5707	**0.0000^a^**
	0.1% triton X-100 vs. 0.3%triton X-100	0.0243	0.002	0.6372	0.3445	0.0034	0.856	0.9504	0.0108
	0.1% triton X-100 vs. 0.6%triton X-100	0.1915	0.006	0.7659	0.1067	0.0246	0.9709	0.0421	**0.0000^a^**
	0.3% triton X-100 vs. 0.6%triton X-100	0.2348	0.0154	0.6123	0.3622	0.5493	0.8848	0.0484	**0.0000^a^**
	0.1% triton X-100 vs. Control	**0.0000^a^**	**0.0001^a^**	0.0019	0.5655	0.0769	0.0368	0.3234	**0.0000^a^**
	0.3% triton X-100 vs. Control	**0.0000^a^**	**0.0001^a^**	0.003	0.9247	**0.0000^a^**	0.0294	0.2974	**0.0000^a^**
	0.6% triton X-100 vs. Control	**0.0000^a^**	**0.0001^a^**	0.0158	0.4649	**0.0000^a^**	0.0353	0.0064	**0.0000^a^**
	10 min SC vs. 20 min SC	0.9586	**0.0000^a^**	0.9587	0.7712	0.0941	0.7663	0.0013	**0.0000^a^**
	10 min SC vs. 30 min SC	0.8407	0.0145	0.1718	0.0243	0.4485	0.8607	0.0051	**0.0001^a^**
	20 min SC vs. 30 min SC	0.8685	**0.0001^a^**	0.2413	0.0271	0.0584	0.6259	0.5158	**0.0000^a^**
	10 min SC vs. Control	**0.0000^a^**	**0.0000^a^**	0.0012	0.1466	**0.0001^a^**	0.0869	0.1699	**0.0000^a^**
	20 min SC vs. Control	**0.0000^a^**	0.0145	0.0012	0.0694	0.0072	0.1318	0.0297	**0.0000^a^**
	30 min SC vs. Control	**0.0000^a^**	**0.0001^a^**	**0.0003^a^**	**0.0007^a^**	**0.0007^a^**	0.058	0.1024	**0.0000^a^**
	10 min UV vs. 20 min UV	0.9628	0.5786	0.4782	0.0717	0.1224	0.6365	**0.0004^a^**	0.3059
	10 min UV vs. 30 min UV	0.7618	**0.0006^a^**	0.2593	0.0237	0.034	0.6893	0.2976	**0.0000^a^**
	20 min UV vs. 30 min UV	0.7042	**0.0003^a^**	0.5665	0.7701	0.4524	0.9676	0.0086	**0.0000^a^**
	10 min UV vs. Control	**0.0000^a^**	0.5786	0.0171	**0.0001^a^**	0.5879	0.0026	0.3347	**0.0000^a^**
	20 min UV vs. Control	**0.0000^a^**	**0.0006^a^**	**0.0005^a^**	**0.0000^a^**	0.0655	**0.0009^a^**	0.0011	**0.0000^a^**
	30 min UV vs. Control	**0.0000^a^**	**0.0003^a^**	**0.0007^a^**	**0.0000^a^**	0.0193	0.0015	0.0416	**0.0000^a^**

Global decellularization groups were compared with the control non-decellularized samples and with the rest of global groups. Specific decellularization groups were compared with controls and with the other specific groups corresponding to the same decellularization agent. All comparisons were carried out by using the Student *t* test except the HE results, which were compared with the Mann-Whitney non-parametric test. Significant *p* values are labeled with “a”, whereas marginally significant *p* values are labeled with “b”.

In the second place, the analysis of residual DNA in the decellularized tissues showed that all global decellularization methods except the NaCl group were able to significantly reduce the DNA content of the tissues as compared with the control (Fig. 1B and Tables 1 and 2). All specific groups except the 1.5 M NaCl, the 20 min SC and the 10 min UV groups had significantly lower DNA than control non-decellularized tissues. The most efficient global groups were SDS (8.63±4.06 ng of DNA per mg of dry weight of decellularized tissue) and triton X-100 (9.70±14.05), which were significantly more efficient than the SC and UV global groups, although statistical significance was not reached when compared with the NaCl global group. No statistical differences were found among the specific concentrations of SDS and triton X-100, whereas some specific concentrations and exposure times of NaCl, SC and UV were different to the other conditions.

Finally, the analysis of tissue weight demonstrated that SI treated with SDS, triton X-100 and SC lost a significant percentage of weight after decellularization (average weight of decellularized tissues was 34.2±14.3%, 24.6±7.7% and 66.4±12.9% of control tissues for the global groups, respectively). Samples decellularized with NaCl or UV did not significantly modify their weight (112.3±24.3% and 91.4±21.9 for the global groups, respectively). No differences were found among the different conditions corresponding to the same decellularization agent.

### Semi-quantitative Analysis of Tissue Structure as Determined by HE Staining and SEM

The semi-quantitative analysis of tissue structure preservation using HE staining evidenced that all global decellularization groups were significantly different to control non-decellularized tissue samples (Tables 1 and 2). No statistical differences were found when the global groups of decellularization agents were compared, although the groups tending to show the best results were the NaCl (1.00, 0.00–2.00), triton X-100 (1.00, 0.00–2.00) and UV (1.00, 1.00–2.00) global groups. The comparison between different concentrations and times of each decellularization agent did not reveal any statistically significant differences for any of the agents. Illustrative images corresponding to the HE staining analysis of control and decellularized tissues are shown in Fig. S3.

**Figure 2:**
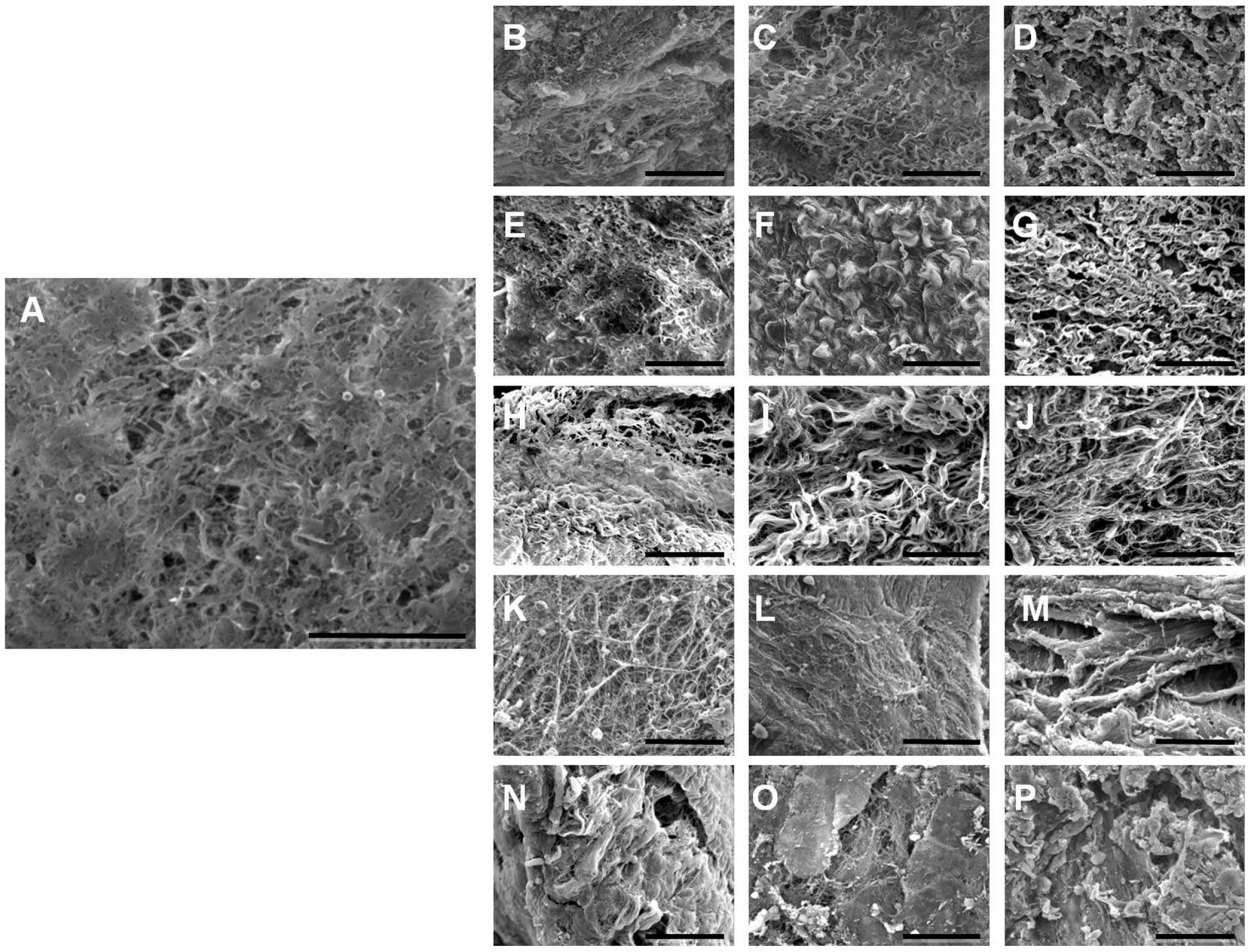
Scanning electron microscopy (SEM) images of control SI samples (A), and tissues decellularized with 1.5 M NaCl (B), 3 M NaCl (C), 5 M NaCl (D), 0.1% SDS (E), 0.3% SDS (F), 0.6% SDS (G), 0.1% triton X-100 (H), 0.3% triton X-100 (I), 0.6% triton X-100 (J), 10 min SC (K), 20 min SC (L), 30 min SC (M), 10 min UV (N), 20 min UV (O) and 30 min UV (P). Scale bar represents 50 µm.

Scanning electron microscopy results (Fig. 2 and Table 1) evidenced that the use of chemical agents as decellularizing solution was associated to better ECM structural organization as compared to physical methods. First, samples corresponding to the 0.1% triton X-100 group had small interfibrillar spaces and a regular and well-arranged fiber mesh, with the best results corresponding to 0.1% triton X-100. Then, NaCl-treated tissues were also able to maintain the ECM structure, except the 5 M NaCl group, which had interfibrillar spaces and low fiber organization. In general, samples decellularized with SDS had some ECM alterations, although 0.1% SDS samples were properly preserved. Finally, samples exposed to physical decellularization agents (SC and UV) exhibited an important increase of the interfibrillar spaces, with an irregular fiber organization and poor ECM structure preservation.

### Quantitative Analysis of the ECM Collagen Fibers as Determined by Picrosirius Staining

**Figure 3:**
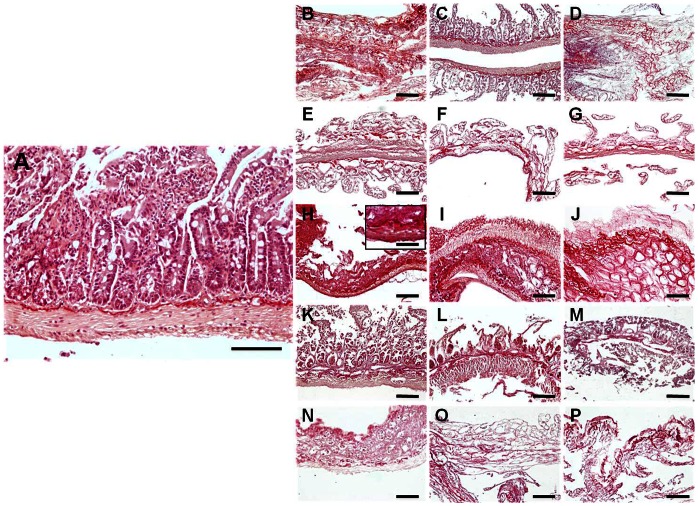
Picrosirius collagen fibers staining of control SI samples (A), and tissues decellularized with 1.5 M NaCl (B), 3 M NaCl (C), 5 M NaCl (D), 0.1% SDS (E), 0.3% SDS (F), 0.6% SDS (G), 0.1% triton X-100 (H), 0.3% triton X-100 (I), 0.6% triton X-100 (J), 10 min SC (K), 20 min SC (L), 30 min SC (M), 10 min UV (N), 20 min UV (O) and 30 min UV (P). All scale bars represent 200 µm, with exception of the insert representing a higher magnification image (scale bar = 800 µm).

Quantification of collagen fibers by Picrosirius staining (Fig. 1C and Fig. 3 and Table 1) showed that the SDS and UV global groups had significantly less collagen staining than control non-decellularized tissues (78.88±3.14% of the control in the SDS group and 73.46±2.79% in the UV group) and that the NaCl (92.24±3.59%), triton X-100 (100.27±2.70%) and SC (87.57±2.99%) global groups. In turn, the picrosirius staining of the NaCl, triton X-100 and SC global groups was similar to the control non-decellularized tissues. For the specific groups, only the highest concentrations of SDS, the 30 min SC group and all UV groups had significantly lower picrosirius signal than control SI. When the specific decellularization conditions were analyzed, the highest intensity values were found in samples treated with 0.1% triton X-100 (102.86±3.29%), although no statistical differences were found with 0.3% and 0.6% triton X-100. Most conditions corresponding to the decellularization agents NaCl, SDS, SC and UV did not differ from the rest of conditions, except for the 0.1% SDS group, which had more staining signal than the 0.3% and the 0.6% SDS groups (Table 2).

### Quantitative Analysis of the ECM Reticular Fibers as Determined by Gomori Reticulin Staining

**Figure 4:**
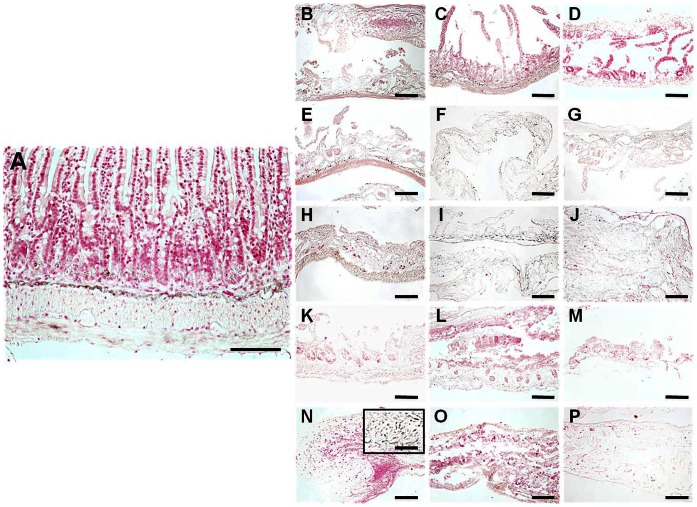
Gomori reticulin staining of control SI samples (A), and tissues decellularized with 1.5 M NaCl (B), 3 M NaCl (C), 5 M NaCl (D), 0.1% SDS (E), 0.3% SDS (F), 0.6% SDS (G), 0.1% triton X-100 (H), 0.3% triton X-100 (I), 0.6% triton X-100 (J), 10 min SC (K), 20 min SC (L), 30 min SC (M), 10 min UV (N), 20 min UV (O) and 30 min UV (P). All scale bars represent 200 µm, with exception of the insert representing a higher magnification image (scale bar = 800 µm).

The Gomori reticulin results demonstrated that the staining intensity was reduced in the NaCl (94.56±10.26% of the control), SDS (94.69±8.55%), triton X-100 (94.91±10.50%) and SC global groups (93.77±2.07%). The only global group that did not change the staining intensity after decellularization was UV (98.03±14.64), which had significantly higher signal than the other four global groups, and did not differ from the control group (Fig. 1D and Fig. 4 and Tables 1 and 2). The results obtained with the different specific UV exposure times were also similar to those of control tissues. Differences were found between some specific concentrations of SDS (0.1% vs. 0.6%).

### Quantitative Analysis of the ECM Glycoproteins as Determined by PAS Staining

**Figure 5:**
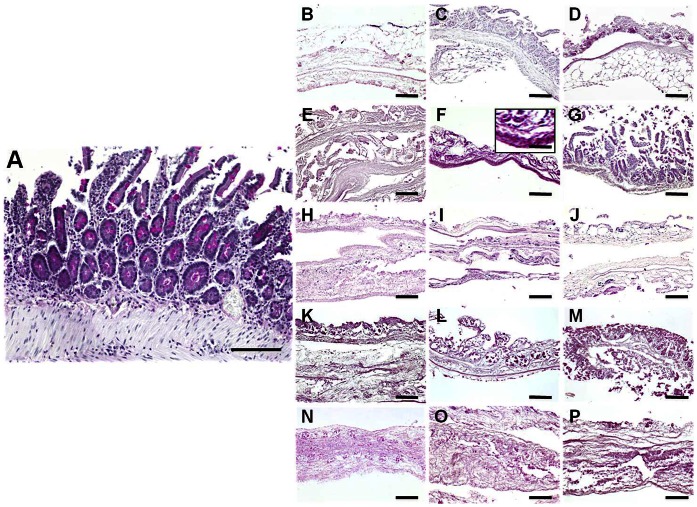
PAS glycoprotein staining of control SI samples (A), and tissues decellularized with 1.5 M NaCl (B), 3 M NaCl (C), 5 M NaCl (D), 0.1% SDS (E), 0.3% SDS (F), 0.6% SDS (G), 0.1% triton X-100 (H), 0.3% triton X-100 (I), 0.6% triton X-100 (J), 10 min SC (K), 20 min SC (L), 30 min SC (M), 10 min UV (N), 20 min UV (O) and 30 min UV (P). All scale bars represent 200 µm, with exception of the insert representing a higher magnification image (scale bar = 800 µm).

As shown in Fig. 1E and Fig. 5 and Tables 1 and 2, PAS staining intensity significantly decreased in the NaCl and UV global groups as compared to control tissues (44.19±9.50% and 66.48±12.89%, respectively), especially the NaCl group, although SDS, triton X-100 and SC global groups were not different to controls (84.34±14.06%, 80.47±9.67% and 82.20±15.98%, respectively). For the specific groups, all NaCl concentrations and the use of UV for 20 min resulted in significantly reduced PAS staining intensity as compared to control non-decellularized tissues. Finally, our results showed that some specific SDS concentrations (0.1% and 0.3%) were associated to higher staining intensities than 0.6% SDS. No differences were found among specific groups of samples treated with the rest of decellularization agents.

### Quantitative Analysis of the ECM Proteoglycans as Determined by Alcian Blue Staining

**Figure 6:**
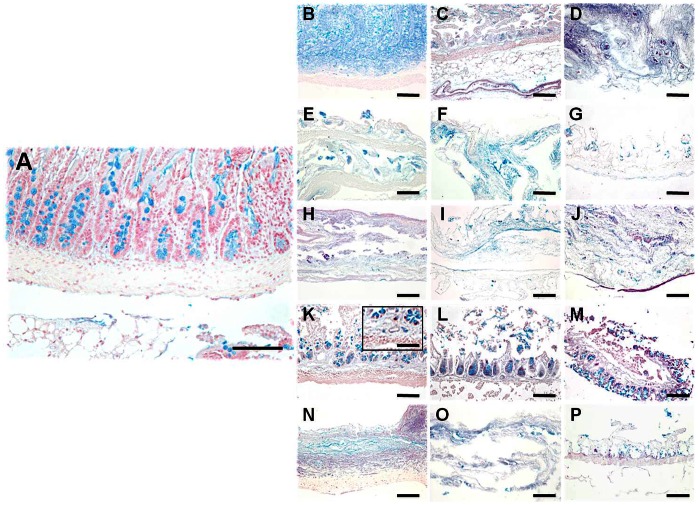
Alcian blue proteoglycan staining of control SI samples (A), and tissues decellularized with 1.5 M NaCl (B), 3 M NaCl (C), 5 M NaCl (D), 0.1% SDS (E), 0.3% SDS (F), 0.6% SDS (G), 0.1% triton X-100 (H), 0.3% triton X-100 (I), 0.6% triton X-100 (J), 10 min SC (K), 20 min SC (L), 30 min SC (M), 10 min UV (N), 20 min UV (O) and 30 min UV (P). All scale bars represent 200 µm, with exception of the insert representing a higher magnification image (scale bar = 800 µm).

The results corresponding to alcian blue staining were very similar among groups (Fig. 1F and Fig. 6 and Tables 1 and 2), with no statistical differences between the control group and any of the global or specific decellularization groups. The histochemical staining intensity was above 96% of the control for all global groups, with no significant differences among groups (97.51±10.49% for NaCl, 97.74±9.53% for SDS, 98.05±8.03% for triton X-100, 98.35±11.48% for SC and 96.27±10.77% for UV). For the specific groups, only the 20 min UV group was significantly different to the 10 min UV group.

### Analysis of the Optical Properties of the Decellularized Tissues

**Figure 7:**
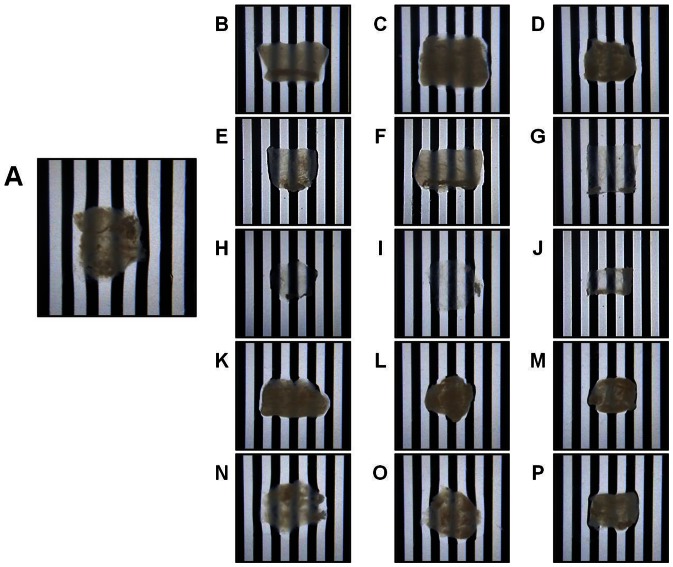
Photographic images of control and decellularized SI samples using a patterned surface to estimate transparency. Images correspond to control SI samples (A), and tissues decellularized with 1.5 M NaCl (B), 3 M NaCl (C), 5 M NaCl (D), 0.1% SDS (E), 0.3% SDS (F), 0.6% SDS (G), 0.1% triton X-100 (H), 0.3% triton X-100 (I), 0.6% triton X-100 (J), 10 min SC (K), 20 min SC (L), 30 min SC (M), 10 min UV (N), 20 min UV (O) and 30 min UV (P).

**Figure 8:**
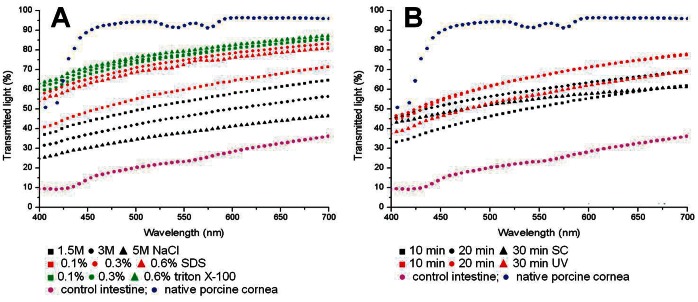
Light transmittance analysis of control and decellularized SI samples. Panel A corresponds to samples decellularized using chemical agents, whereas results of the application of physical decellularization methods are shown in panel B. Non-decellularized intestine and native porcine cornea are shown as controls.

As shown in Fig. 7, tissues decellularized with the different protocols described in this work showed different gross transparency levels, with the highest gross transparency corresponding to the triton X-100 and SDS decellularization groups. In addition, the analysis of the spectral distribution of the transmitted light through decellularized samples (Fig. 8) demonstrated that all global and specific groups had significantly higher transmittance levels than control non-decellularized tissues for all analyzed wavelengths (p<0.001). The highest light transmittance values corresponded to the triton X-100 and SDS global groups. Both the triton X-100 and SDS global groups were significantly higher than NaCl global group, and the triton X-100 global group was higher to the UV global group. However, non-significant differences were found with the SC global group (Table 2). For the specific decellularization groups, statistical differences were detected when different conditions were compared for the same decellularization agent, except for 0.1% vs. 0.3% triton X-100 and 10 min vs. 20 min UV. Independently of the decellularization treatment used, all samples displayed lower transmittance values than the native porcine cornea (values close to 95%), but differences were not statistically significant.

## Discussion

Decellularized tissues and organs recently emerged as efficient alternatives to the use of hydrogels and synthetic biomaterials for corneal replacement [13]. Scaffolds generated by decellularization proved to be useful for the generation of different tissues both in vitro and in vivo, and some preclinical and clinical studies suggest that these scaffolds may be clinically useful [27].

During the decellularization process, an ideal decellularization agent should be able to efficiently remove all cells and cellular components from the decellularized tissue without altering the histological structure and chemical composition of the native ECM [17]. The ECM plays an important role in the physiology of the cells and therefore, in the function of the tissue itself. In fact, it has been demonstrated that the ECM provides the means by which cells communicate [29], divide [18] and differentiate [30]. In addition, the ECM is the host structure supporting blood, lymphatic vessels and innervations, thus allowing cell nutrition and oxygen diffusion [29]. In the case of the human cornea, several ECM components are essential for the correct physiology and homeostasis of this organ. The human cornea stroma is mainly composed by a type-I collagen fiber mesh with keratocytes and several growth factors, glycoproteins, proteoglycans and signals that are essential for cell and tissue development. In fact, previous reports suggest that an effective way to reproduce native corneal stromal tissue is the use of scaffolds enriched in growth factors [31]. In turn, the ECM of the small intestine consists of different types of collagen fibers, especially type-I collagen and reticular fibers, with different glycoproteins and proteoglycans, and preservation of these components could favor cornea regeneration. Though the spatial orientation of the collagen fibers is more arranged in the cornea stroma than in SI, the chemical composition of both tissues may be similar and suggests that cornea tissues could be reproduced in the laboratory by using decellularized SI scaffolds, which are much more abundant and easy to obtain than cornea tissues.

In the present work, we have evaluated the accuracy of the most commonly used chemical and physical decellularization methods on small intestine tissues by determining tissue structure, cell removal, ECM components preservation and optical properties in order to optimize a decellularization method for future cornea reconstruction. In this regard, our results first showed that all decellularization agents were able to eliminate most cell nuclei from the tissues, especially the detergent SDS, although suboptimal decellularization levels were found in tissues treated with NaCl. Moreover, the analysis of residual tissue DNA reveled that only the decellularization agents SDS and triton X-100 were capable of efficiently removing most host DNA from the SI (less than 50 ng of residual dsDNA per mg of dry weight of tissue, as recommended by Crapo *et al.* [18]. These results suggest that both chemical detergents are very efficient decellularization agents, resulting in very low residual DNA levels regardless the concentration used. This statement is confirmed by the fact that tissues decellularized with SDS or triton X-100 lost a significant percentage of tissue weight upon decellularization, implying that most cells and other tissue components may have been efficiently removed. Ionic and non-ionic detergents have been reported to solubilize cell membranes and dissociate DNA and proteins [18]. In fact, previous reports demonstrated that these agents are able to remove most cellular material from porcine bone [32], thoracic aorta [33], rabbit intrasynovial tendons [34] and also from small intestine [35]. The use of osmotic chemical agents (NaCl) and physical methods (SC and UV) was less efficient for cell and DNA removal than SDS and triton X-100. However, some specific conditions of these agents could have potential usefulness for tissue decellularization.

On the other hand, preservation of the structure and chemical composition of the ECM of decellularized tissues is one of the most important requisites of a decellularization protocol. The ECM consists of a group of fibrillar components that is the main responsible of the structural integrity of the tissue (mainly collagen, reticular and elastic fibers) and two mayor non-fibrillar components (glycoproteins and proteoglycans). Non-fibrillar ECM components play key roles in cell-cell interaction, cell adhesion, proliferation migration and response, and are essential for the maintenance of the 3D structure and hydration level of the tissue [36]. Due to their crucial function, it is very important that all structural and functional ECM molecules be preserved for further clinical use of the decellularized tissues. For this reason, it is necessary to optimize all decellularization protocols for each tissue type and for every application. In this regard, our analysis demonstrated that all physical and chemical decellularization protocols resulted in certain degree of morphological tissue structure alteration, with no differences among protocols, although chemical agents seemed to be associated to better ECM structural organization than physical protocols as determined by SEM. Interestingly, samples decellularized with triton X-100 showed proper collagen fibers structure and organization, suggesting that this highly-efficient decellularizing agent could preserve tissue ECM structure. Of course, one possibility is that the use of aqueous solutions during the decellularization process could give rise to tissue swelling and cause structural changes in tissue architecture that are not directly driven by the decellularization agent, but by the vehiculating aqueous solution.

Regarding the collagen fibers -mayor fibrillar components of the cornea and SI ECM-, our findings demonstrated that NaCl, triton X-100 and SC were able to decellularize SI samples while preserving the staining intensity of these fibers, with the best results corresponding to triton X-100. In contrast, the use of SDS (except 0.1% concentrations) and UV was associated to a significant loss of collagen fibers picrosirius staining. Previous works by our group showed that cornea decellularization with SDS resulted in partially disorganized collagen fibers [13], although Gilbert *et al.* [17] affirms that collagens are resistant to ionic detergents. Zou and Zhang [33] evidenced that the triton X-100 treatment is effective for maintaining the mayor structure of the elastin and collagen network in the ECM on porcine thoracic aortas. Due to its anionic composition, triton X-100 should have very few impact on protein structure [37], even though few reports state that it could damage the collagen architecture of porcine tendons [38]. The results of the present work indicate that triton X-100 could adequately protect the fibrilar ECM elements and generate a decellularized scaffold with the most suitable biological properties. Taken together, all these results suggest that the agent able to more efficiently preserve the fibrillar ECM components could be triton X-100. This agent should therefore be used when preservation of the ECM fiber mesh is crucial for the regeneration of the tissue, as it is the case of the human cornea. Interestingly, decellularization protocols based on NaCl were also able to preserve high levels of collagen staining in decellularized SI samples. NaCl has been extensively used as a chemical decellularization agent in dermis [39] and lung [40]. Although the exact mechanism by which this agent is able to decellularize is not well known, Gilbert et al. [37] stated that NaCl solutions may induce an osmotic shock that could trigger the rupture of the cell in the tissue. However, Richter *et al.* [41] reported that Na and Cl ions could be able to cross the membrane by controlled channels with no signals of cell disruption, but a hypertonic environment could extract water from the cells and modify the cell volume, which could alter the macromolecular content and induce cell death. Collagen fibers tend to crosslink when they are dehydrated and the fibers are brought closer together [38], allowing the formation of amide bonds between adjacent amine and carboxyl groups. Most likely, the NaCl concentrations used in the present work would not generate these chemical changes and the collagen fibers would be efficiently preserved.

In addition, all decellularization agents preserved more than 93% of the reticular fiber staining of the native tissues, although only UV-treated samples had similar staining levels than control tissues. Fibrillar proteins have been reported to be damaged by UV exposition [42], which could induce interfibrillar cross-linking on porcine valves [43]. However, our findings evidenced that UV exposition did not significantly affect the reticular fibers staining intensity of the SI. Although reticular fibers are important components of the fibrillar ECM along with collagen fibers [44], their different spatial 3D distribution, packaging and molecular structure could make them more sensitive to chemical agents and SC than to UV exposition.

Regarding to the non-fibrillar components of the ECM, our analysis of glycoprotein staining revealed that several methods (SDS, triton X-100 and SC) were able to efficiently preserve these ECM components. The use of SDS and triton X-100 detergents demonstrated to be safe for glycoprotein preservation after decellularization, probably due to the chemical nature of these components of the ECM. Although the best results were found for 0.1% and 0.3% SDS, high staining levels were found in samples treated with SC. This physical method was previously found to be useful to disrupt cell membranes, release cell contents and facilitate the subsequent rinsing and removal of cell remnants from the ECM [27], but it may be very inefficient when used separately. As stated above, SC was able to preserve collagen fibers along with glycoproteins, although our results demonstrated that this technique was not as efficient as other methods to preserve other fibrillar and extrafibrillar ECM components that may be more sensitive to the mechanical stimuli generated by SC method, especially during long time periods. On the other hand, the alcian blue staining results showed that all decellularization agents were able to preserve most ECM proteoglycans at all concentrations and times assayed here. It is probable that the 3D structure of these components and their strong attachment to the rest of ECM components prevent their degradation and release during the decellularization process and supports the use of any physical or chemical decellularization method. Taken together, these results suggest that non-fibrillar ECM components of the SI may be efficiently preserved by using SDS, triton X-100 or SC methods.

Once the fibrillar and non-fibrillar components of the ECM were evaluated, in this work we analyzed the optical behavior of all decellularized SI tissues. With a view on the subsequent use of the decellularized tissues for use in cornea reconstruction, it is very important to analyze the optical properties of the decellularized SI and to guarantee that these scaffolds display proper optical behavior and light transmittance. Although the transparence levels of the decellularized tissues never became similar to those of the native cornea, all decellularization protocols were able to significantly increase the tissue transmittance as compared to the control SI, reaching transparence levels that were near the native cornea for some chemical agents, especially for triton X-100 and SDS. The use of other decellularization agents (NaCl, SC and UV) could induce some chemical or physical modifications in the ECM structure that could impair light transmission through the tissues.

In conclusion, our results point out the usefulness of decellularized intestinal grafts for corneal tissue engineering with the advantages of easy obtaining and accessibility. According to our results, the most efficient decellularization agent may be the SDS and triton X-100 detergents. Both agents could efficiently remove most cell nuclei and residual DNA, while the non-fibrillar ECM components and the tissue optical properties were preserved after the decellularization process. In addition, the use of triton X-100 was able to maintain the collagen intensity and tissue structure, suggesting that this specific decellularization agent could be safely used for efficient decellularization of SI tissues for cornea TE. Further works should confirm these results and determine if the use of combined decellularization protocols could improve the results obtained with each agent used separately.

## Supporting Information

Figure S1 **Graphical examples for the scale of tissue structure preservation used in this work.** Control sample (A); decellularized SI with highly organized ECM scoring 0 (B); decellularized SI with low levels of disorganization of the ECM scoring 1 (C); decellularized SI with intermediate levels of disorganization of the ECM scoring 2 (D); and decellularized SI with high levels of disorganization of the ECM scoring 3 (E). All samples were stained with HE. Scale bar represents 200 µm.
(TIF)

Figure S2 **Illustrative examples for cell nuclei evaluation using DAPI staining. Control (A); 5 M NaCl (B); 0.6% SDS (C); 0.1% triton X-100 (D); 10 min SC (E) and 10 min UV (F). Scale bar represents 200 µm.**
(TIF)

Figure S3 **HE staining of control SI samples (A), and tissues decellularized with 1.5 M NaCl (B), 3 M NaCl (C), 5 M NaCl (D), 0.1% SDS (E), 0.3% SDS (F), 0.6% SDS (G), 0.1% triton X-100 (H), 0.3% triton X-100 (I), 0.6% triton X-100 (J), 10 min SC (K), 20 min SC (L), 30 min SC (M), 10 min UV (N), 20 min UV (O) and 30 min UV (P).** Scale bar represents 200 µm.
(TIF)
