# Supplementary figures and images for: Evaluation of Small Intestine Grafts Decellularization Methods for Corneal Tissue Engineering
Source: PLoS One. 2013 Jun 14;8(6):e66538. doi: 10.1371/journal.pone.0066538 (PMC3682956; doi:10.1371/journal.pone.0066538)

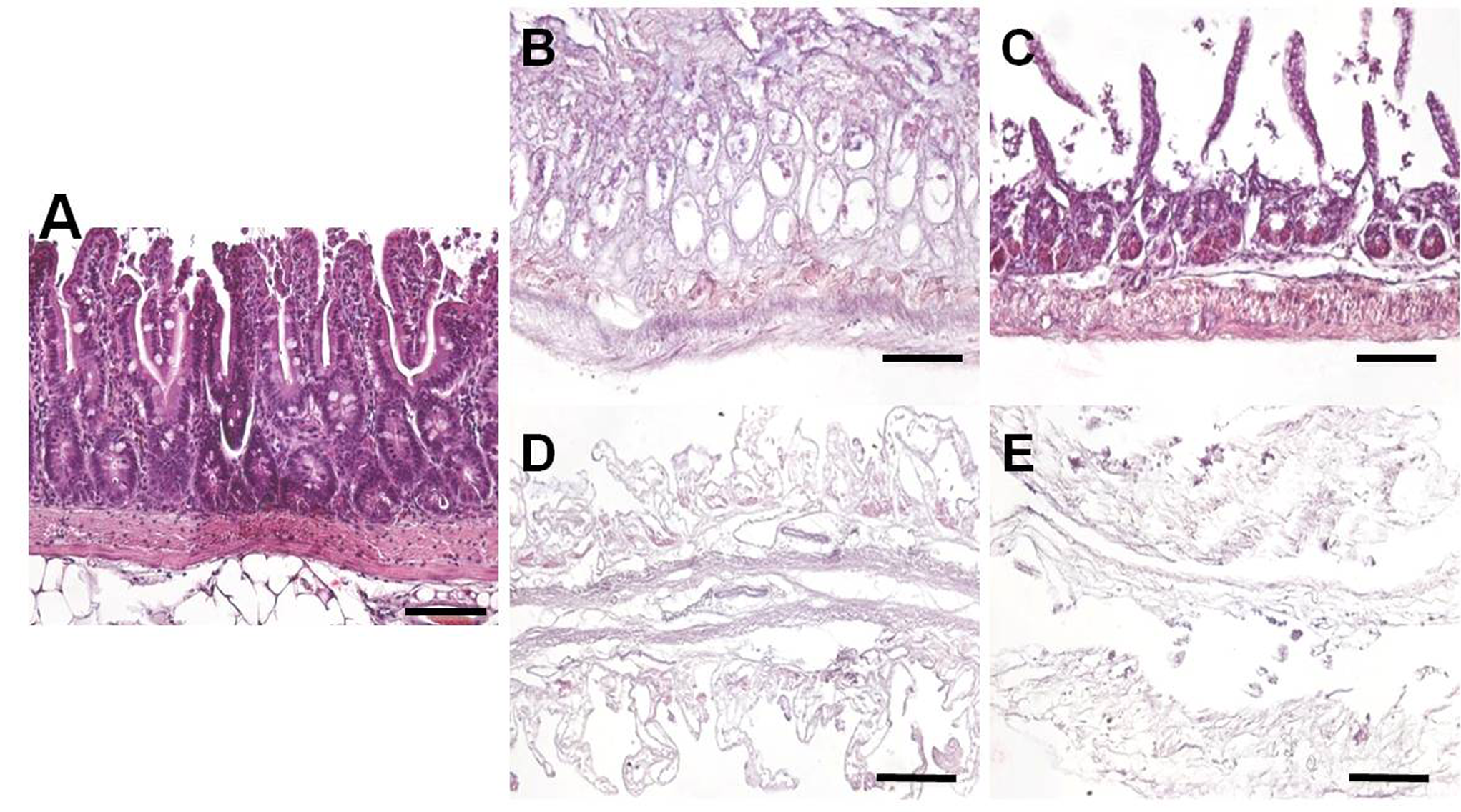

Supplement: Figure S1 — Graphical examples for the scale of tissue structure preservation used in this work. Control sample (A); decellularized SI with highly organized ECM scoring 0 (B); decellularized SI with low levels of disorganization of the ECM scoring 1 (C); decellularized SI with intermediate levels of disorganization of the ECM scoring 2 (D); and decellularized SI with high levels of disorganization of the ECM scoring 3 (E). All samples were stained with HE. Scale bar represents 200 µm. (TIF) [file pone.0066538.s001.tif]

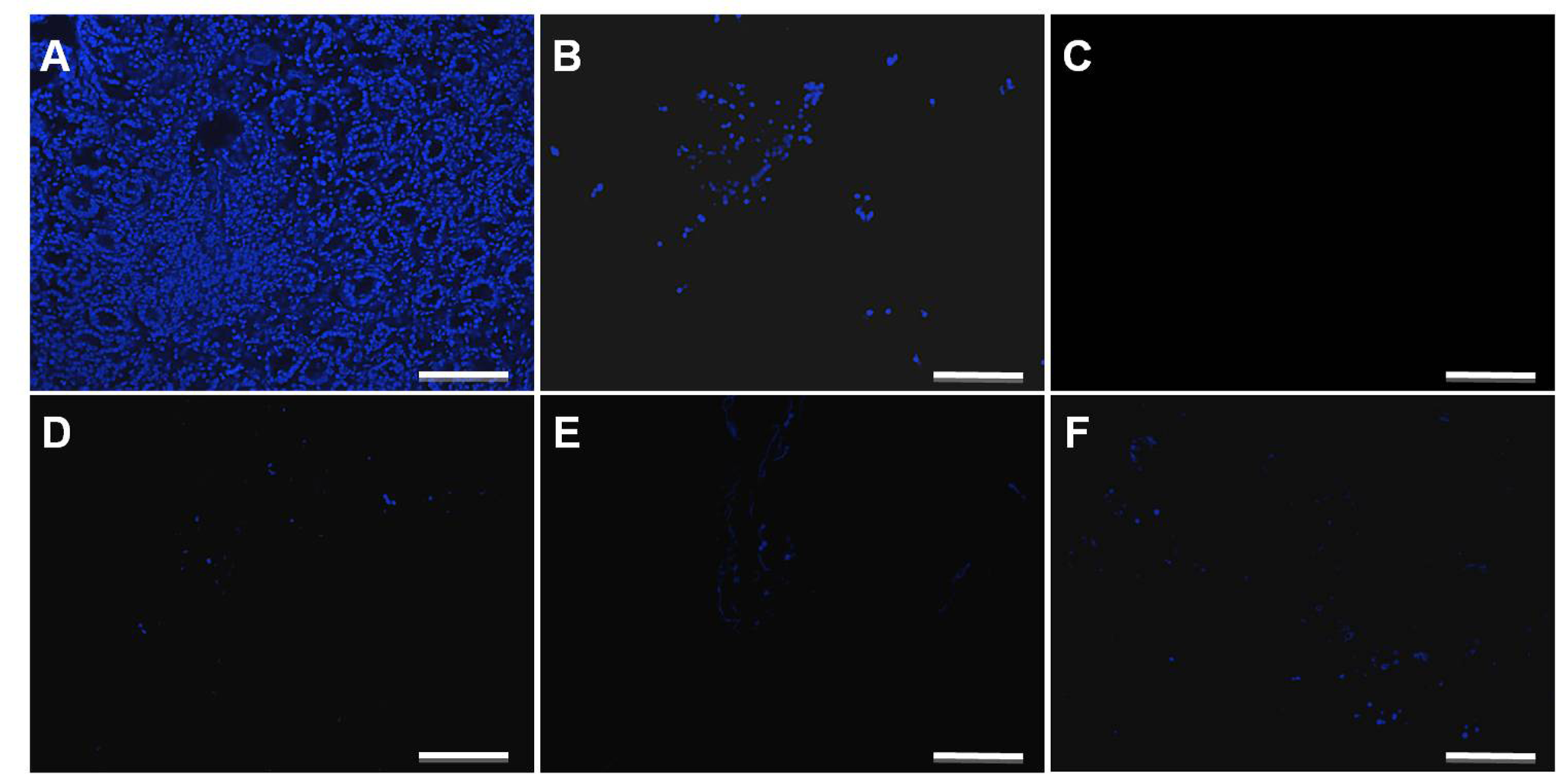

Supplement: Figure S2 — Illustrative examples for cell nuclei evaluation using DAPI staining. Control (A); 5 M NaCl (B); 0.6% SDS (C); 0.1% triton X-100 (D); 10 min SC (E) and 10 min UV (F). Scale bar represents 200 µm. (TIF) [file pone.0066538.s002.tif]

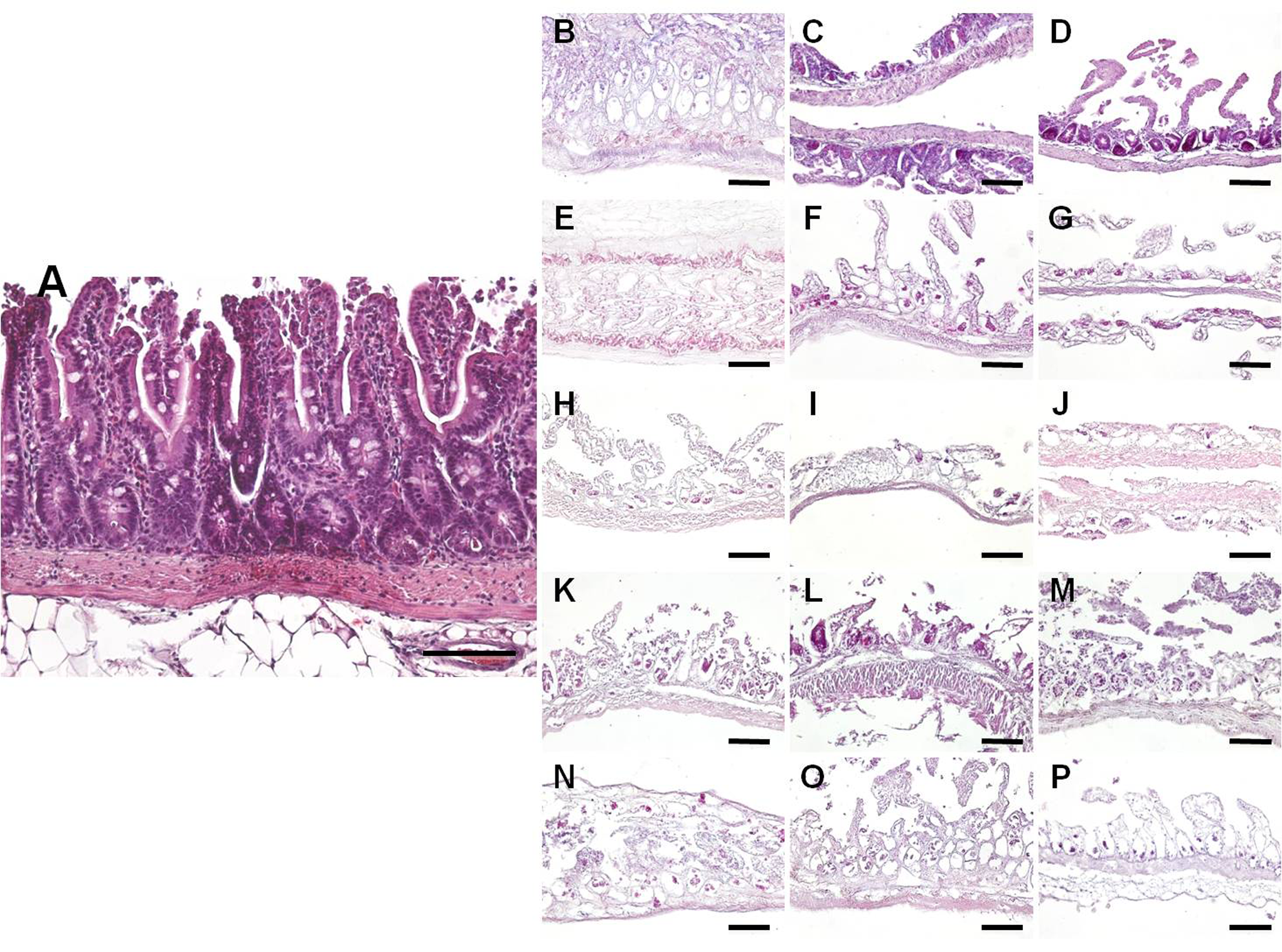

Supplement: Figure S3 — HE staining of control SI samples (A), and tissues decellularized with 1.5 M NaCl (B), 3 M NaCl (C), 5 M NaCl (D), 0.1% SDS (E), 0.3% SDS (F), 0.6% SDS (G), 0.1% triton X-100 (H), 0.3% triton X-100 (I), 0.6% triton X-100 (J), 10 min SC (K), 20 min SC (L), 30 min SC (M), 10 min UV (N), 20 min UV (O) and 30 min UV (P). Scale bar represents 200 µm. (TIF) [file pone.0066538.s003.tif]
